# Cancer-Preventive Role of Bone Marrow-Derived Mesenchymal Stem Cells on Colitis-Associated Colorectal Cancer: Roles of Gut Microbiota Involved

**DOI:** 10.3389/fcell.2021.642948

**Published:** 2021-06-04

**Authors:** Ruohang He, Chaoqun Han, Ying Li, Wei Qian, Xiaohua Hou

**Affiliations:** Division of Gastroenterology, Union Hospital, Tongji Medical College, Huazhong University of Science and Technology, Wuhan, China

**Keywords:** Mesenchymal stem cells, gut microbiota, inflammatory bowel disease, colitis-associated colorectal cancer, RNA sequencing

## Abstract

**Background:**

Mesenchymal stem cells (MSCs) treatment showed promising results in inflammatory bowel disease in both rodent models and patients. Nevertheless, previous studies conducted conflicting results on preclinical tumor models treated with MSCs concerning their influence on tumor initiation and progression. This study is designed to demonstrate the role of bone marrow-derived MSCs and the potential mechanism in the colitis-associated colon cancer (CAC) model.

**Methods:**

Bone marrow-derived MSCs were isolated from green fluorescent protein-transgenic mice, cultured, and identified by flow cytometry. Azoxymethane and dextran sulfate sodium were administrated to establish the CAC mouse model, and MSCs were infused intraperitoneally once per week. The mice were weighed weekly, and colon length, tumor number, and average tumor size were assessed after the mice were killed. MSC localization was detected by immunofluorescence staining; tumor cell proliferation and apoptosis were measured by immunohistochemistry staining of Ki-67 and terminal deoxynucleotidyl transferase deoxyuridine triphosphate nick end labeling assay, respectively. The colonic tumor tissues were isolated for RNA-seq, and fecal samples were collected for 16S ribosomal RNA sequencing of the microbiome.

**Results:**

After injection intraperitoneally, MSCs migrated to the intestine and inhibited the initiation of colitis-associated colorectal cancer. This inhibition effect was marked by less weight loss, longer colon length, and reduced tumor numbers. Moreover, MSCs reduced tumor cell proliferation and induced tumor cell apoptosis. Furthermore, MSCs could inhibit chronic inflammation assessed by RNA-sequencing and promote gut microbiome normalization detected by 16S ribosomal RNA sequencing.

**Conclusion:**

The results proved that MSCs could migrate to the colon, inhibit chronic inflammation, and regulate gut microbiome dysbiosis to suppress the development of CAC.

## Introduction

Colorectal cancer (CRC) is the third leading cause of cancer and the second-highest cause of cancer mortality globally (Bray et al., 2018). A key driver of the progression of CRC is chronic inflammation (Keum and Giovannucci, 2019). Patients with inflammatory bowel disease (IBD), referred to as a chronic inflammatory disease, have an increased risk for a type of CRC known as colitis-associated colon cancer (CAC) (Chumanevich et al., 2010; Jess et al., 2012). The risk of developing CAC can be effectively reduced by anti-inflammatory medications (Van Staa et al., 2005; Vendramini-Costa and Carvalho, 2012); however, long-term use is limited because of their life-threatening adverse effects (Wang and DuBois, 2013); therefore, there is an urgent need to explore new anti-inflammatory therapeutic approaches to cancer.

Mesenchymal stem cells (MSCs), which are multipotent stem cells, possess the capacity for long-term self-renewal and multidirectional differentiation (Sekiya et al., 2002). Moreover, MSCs can migrate to an inflammatory or tumor site and display profound immune-modulatory (Bouffi et al., 2010; Chiossone et al., 2016; Gao et al., 2016; Chow et al., 2017; Wheat et al., 2017). It has been reported that intravenous or intraperitoneal injection of MSCs could significantly reduce colonic inflammation in both colitis rodent and IBD patients (Liang et al., 2011; Anderson et al., 2013; Park et al., 2015; Lee et al., 2016; Cao et al., 2017). However, there have been conflicting results on preclinical tumor models treated with MSCs concerning their influence on tumor development. We summarized all the studies relating to MSCs and colon cancer (Table 1; Shinagawa et al., 2010; Liu et al., 2011; Tsai et al., 2011; De Boeck et al., 2013; Huang et al., 2013; Chen et al., 2014; Mele et al., 2014; Nasuno et al., 2014; Rhyu et al., 2015; Tang et al., 2015; Wang et al., 2015; Widder et al., 2016) and found that studies demonstrating that MSCs promote tumors were based on the subcutaneous implantation of human cancer cell lines into an immunodeficient nude mouse model, which is not representative of the clinicopathology. Regarding this, the azoxymethane (AOM)/dextran sulfate sodium (DSS) mouse model, which closely mimics the mechanisms of human CAC, is an appropriate animal model for studying preclinical insights on the impact of molecular markers (De Robertis et al., 2011). Although several studies had already demonstrated that MSCs could be therapeutically effective in this CAC mouse model (Chen et al., 2014; Nasuno et al., 2014; Tang et al., 2015), Chen et al. (2014) found that MSCs inhibited tumor number through IL-6-STAT3 signaling; Tang et al. (2015) showed that MSCs suppressed the development of CAC through regulating the differentiation of Treg cells *via* Smad2, whereas Nasuno et al. (2014) demonstrated that MSCs inhibited tumor initiation by affecting tumor cell-cycle machinery; however, the protective mechanisms have not been fully defined.

**TABLE 1:** Key finding from studies using MSCs to treat colon cancer.

Author	Isolation	Tumor model	Findings
**Tumor promoting**			
Wang et al., 2015	Human BMD-MSC	Subcutaneous xenograft ± coinjected MSC	Secretion of IL-8 by MSCs promotes tumor growth
Widder et al., 2016	Human BMD-MSC	Subcutaneous xenograft ± coinjected MSC	Secretion of β1-integrity by CRC participates in the effect of MSCs
Mele et al., 2014	Human BMD-MSC	Subcutaneous xenograft ± coinjected MSC	MSCs triggered EMT, mediated by TGF-β expressed on MSCs
De Boeck et al., 2013	Human BMD-MSC	Subcutaneous xenograft ± coinjected MSC	MSCs affected tumor initiation and growth
Huang et al., 2013	Not specified	Subcutaneous xenograft ± coinjected MSC	MSCs increased tumor growth rate and angiogenesis
Liu et al., 2011	Mouse BMD-MSC	Subcutaneous xenograft ± coinjected MSC	MSCs enhanced tumor growth
Tsai et al., 2011	Human BMD-MSC	Subcutaneous xenograft ± coinjected MSC	MSCs derived IL-6 promoted tumor formation
Shinagawa et al., 2010	Human BMD-MSC	Orthotopic colon cancer ± coinjected MSC	MSCs promoted tumor growth and liver metastasis
**Tumor inhibiting**			
Chen et al., 2014	Mouse BMD-MSC	AOM/DSS ± MSC	MSCs inhibited tumor number through IL-6-STAT3 signaling
Tang et al., 2015	Umbilical cord blood	AOM/DSS ± MSC	MSCs inhibited tumor initiation by inhibiting inflammatory cytokine production
Nasuno et al., 2014	Rat BMD-MSC	AOM/DSS ± MSC	MSCs inhibited tumor initiation by affecting tumor cell-cycle machinery
Rhyu et al., 2015	Rat BMD-MSC	Subcutaneous allograft ± MSC	MSCs inhibited the outgrowth of the rat colon carcinoma and induced greater monocyte infiltration

*BMD-MSC, bone marrow-derived mesenchymal stem cell; CRC, colorectal cancer; EMT, epithelial to mesenchymal transition; AOM, azoxymethane; DSS, dextran sulfate sodium.*

Gut microbes are involved in the intestinal defense function and the immune system maturation, whereas gut microbiome dysbiosis participates in the pathogenesis of IBD and CRC (Jostins et al., 2012; Flemer et al., 2018). Soontararak et al. (2018) demonstrated that MSCs could ameliorate colonic inflammation and gut microbiome dysbiosis in mouse IBD models. So, we hypothesized that MSCs could ameliorate CAC through modulating both immunity and the gut microbiome.

In this experiment, we aimed to elucidate the cancer-preventive role and mechanisms of MSCs in a CAC mouse model induced with AOM and DSS. We found MSC administration attenuated adenoma initiation, decreased chronic inflammation, and regulate gut microbiota dysbiosis.

## Materials and Methods

### Mice

Male C57BL/6 mice (6 weeks old) were purchased from Beijing Huafukang Biotechnology Co., Ltd. (Beijing, China). The animals were kept in specific pathogen-free conditions at room temperature 22 ± 2°C and 55 ± 5% relative humidity under a light/dark cycle for 12 h. Water and feed were supplied *ad libitum* during the whole experiment. The current experiment was approved by the Animal Care and Use Committee of Tongji Medical College of Huazhong University of Science and Technology (permission number: 2016-0057). All procedures were conducted in concordance with the Declaration of Helsinki and the Chinese Ministry of Health (document no. 55, 2001).

### Animal Treatment

Mice were kept for 1 week without any procedure and randomly assigned to three groups (*n* = 10/group): (1) control group (Con), (2) AOM/DSS group (AD), and (3) MSC group [AME, AOM/DSS treatment, and MSC injection (2 × 10^6^ per mice, intraperitoneal)]. To establish AOM- and DSS-induced CAC mouse model (Vendramini-Costa and Carvalho, 2012), 10 mg/kg AOM (Sigma-Aldrich, St. Louis, MO, United States) were intraperitoneally injected at the beginning of week 0, and 2.5% DSS (molecular weight 36–50 kDa; MP Biomedicals, Solon, OH, United States) was then dissolved in their drinking water every day for 1 week at the start of week 1, followed by normal drinking water for next 2 weeks; thus, DSS was administrated at weeks 1–2, 4–5, and 7–8 for the whole experiment. The MSC [2 × 10^6^ cells in 0.2-ml phosphate-buffered saline (PBS)] was injected once per week during the study, whereas 0.2-ml pure PBS was injected in the control group and AOM/DSS group mice accordingly. Body weight was measured and recorded weekly. The mice were killed after anesthesia at week 10. After colon length was recorded, colons were incised longitudinally; fecal contents were collected and then washed for further analysis. The tumor number was counted, and the tumor size was measured using a caliper. Tumor tissues were fixed in 4% paraformaldehyde and subsequently used for hematoxylin and eosin (H&E), immunohistochemistry, and immunofluorescence. The other tissues were stored for further analysis.

### Culture and Identification of Mesenchymal Stem Cells

Male C57BL/6 green fluorescent protein (GFP)-transgenic mice (3–4 weeks old) were purchased from Cyagen Biosciences (Cyagen Biosciences Inc., Guangzhou, China). Bone marrow-derived MSCs were isolated from the GFP-transgenic mice as previously described (Lin et al., 2020). Briefly, mice were killed after anesthesia, soaked in 75% ethanol for 3 min. Femurs and tibias were separated and removed of muscle and connective tissues; bone marrow was flushed out by using a 1-ml sterile syringe with a complete culture medium consisting of low glucose Dulbecco’s modified Eagle’s medium (Gibco, Carlsbad, CA, United States) with 10% fetal bovine serum (Gibco^®^ Cell Culture, Melbourne, VIC, Australia) and 1% penicillin/streptomycin (Gibco, Carlsbad, CA, United States). The cell suspension was centrifuged at 300 × *g* for 5 min, then removed the supernatant and resuspended the cells with the complete culture medium mentioned earlier and plated in T25 cell culture dishes (Nest, Shanghai, China). The supernatant was changed every other day to discard the non-adherent cells. When adherent cells reached more than 80%, 0.25% trypsin–ethylenediaminetetraacetic acid solution (Gibco, Carlsbad, CA, United States) was used to digest cells for 5 min, and collected cells were subcultured. Cells at three to six passages were used for the subsequent experiments.

To identify the surface markers of MSCs obtained earlier, flow cytometry (FCM) analysis was performed. Briefly, cells were collected and washed by PBS two times. Cells were then incubated with fluorescein isothiocyanate-conjugated anti-mouse Sca-1, CD11b, CD45, PE-conjugated CD73, CD90, and APC-conjugated CD44, CD105 (BD Bioscience, NJ, United States) in the dark for 40 min at 4°C. To detect the GFP expression of MSCs, normal MSCs isolated from male C57BL/6 mice were used as control. Cells were washed and resuspended in 100-μl PBS; then, cells were examined using a flow cytometer (BD PharMingen, San Diego, CA, United States). One thousand viable events were collected and analyzed using FlowJo V10 software (Tree Star, Ashland, OR, United States).

### Hematoxylin and Eosin Staining

Colonic specimens were immersed in 4% paraformaldehyde for 24 h, then embedded in paraffin and dehydrated in ethanol using standard procedures (Hong et al., 2010); 5-μm sections were stained with H&E and examined and photographed with a light microscope.

### Immunohistochemistry

Paraffin-embedded sections (5 μm) were deparaffinized and rehydrated through graded alcohols; antigen heat retrieval was conducted in citrate buffer using a pressure cooker and then cooled down to room temperature; hydrogen peroxide solution was then used to block endogenous peroxidase. The slides were incubated overnight with an antibody against mouse Ki67 (1:200, Abcam, Cambridge, MA, United States) at 4°C. Wash the slides with PBS, horseradish peroxidase-conjugated secondary antibody was added to the slides and co-incubated for 2 h; the slides were then incubated for 10 min using DAB kit following the manufacturer’s instructions (Boshide, Wuhan, China) and counterstained with hematoxylin for 2 min. Sections were observed and photographed using a light microscope.

### Terminal Deoxynucleotidyl Transferase Deoxyuridine Triphosphate Nick End Labeling Assay

The terminal deoxynucleotidyl transferase deoxyuridine triphosphate nick end labeling (TUNEL) method was used to detect tumor cellular apoptosis in paraffin-embedded sections (5 μm). TUNEL apoptosis detection kit (Roche, Indianapolis, IN, United States) was applied following the manufacturer’s instruction, and the slides were examined and photographed by fluorescence microscope.

### Immunofluorescence Staining

Paraffin-embedded sections (5 μm) were dewaxed and rehydrated through graded alcohols; antigen heat retrieval was conducted in citrate buffer using a pressure cooker and then cooled down to room temperature; 10% donkey serum was then used to block endogenous antigen for 30 min. The slides were incubated overnight with an antibody against mouse GFP (1:200, Abcam, Cambridge, MA, United States) at 4°C. The slides were washed with PBS, incubated with Alexa Fluor 488 conjugated donkey anti-mouse secondary antibodies (Antigen Biotech Co., Ltd., Wuhan, China) for 1 h, and 4′,6-diamidino-2-phenylindole was used to stain nuclei. Images were photographed by fluorescence microscope.

### RNA Sequencing

The colonic tumor tissues of AOM/DSS and MSC groups were collected and flash-frozen in a −80°C freezer. Total RNA extraction and RNA sequencing were performed by Shenzhen BGI Institute (BGI-Shenzhen, China) using the BGIseq500 platform. SOAPnuke (v1.5.2) was used to filter data, and high-quality reads were aligned to the mice reference genome performed by Bowtie2 (v2.2.5). The expression levels of each gene were normalized to fragments per kilobase million (FPKM). DEGSeq2 (v1.4.5) was used to analyze differential expression gene (DEG) analysis with a *Q* value ≤ 0.05; pheatmap (v1.0.8) was used to draw heatmap according to FPKM in different samples. Then, the Kyoto Encyclopedia of Genes and Genomes (KEGG) enrichment analysis of DEGs was conducted by Phyper based on the hypergeometric test. *Q* value with a strict threshold (*Q* value ≤ 0.05) by Bonferroni was used to correct the significant levels of terms and pathways.

### Enzyme-Linked Immune Sorbent Assay Analysis

Serous concentrations of tumor necrosis factor-α (TNF-α), interleukin (IL)1β, and IL6 were measured by enzyme-linked immune sorbent assay (eBioscience, Thermo Fisher Scientific, United States) according to the manufacturer’s manuals. The absorbance was obtained at relative nanometer wavelength using a microplate reader (BioTek Instruments, Inc., Winooski, VT, United States).

### 16S Ribosomal RNA Sequencing of Fecal Microbiota

The fecal contents were collected after the killing of mice for further analysis. Total fecal DNA extraction and 16S ribosomal RNA (rRNA) sequencing were conducted by the GENEWIZ Institute (Suzhou, China) using the Illumina MiSeq platform. QIIME (v1.9.1) was applied to filter the sequencing data, whereas Vsearch (v1.9.6) was used to cluster the high-quality sequences with a 97% read identity into operational taxonomic units. To determine differences of fecal microbiota between the AOM/DSS and MSC groups, we analyzed the α diversity and β diversity by QIIME. Chao1 and Simpson indices were assessed to determine α diversity, which refers to the diversity within sample community species richness. Unweighted UniFrac distances were applied to characterize β diversity, which represents dissimilarity among different treatment groups. Principal coordinate analysis (PCoA) plot and analysis of similarities (ANOSIM) were used to calculate the β diversity visually. Linear discriminant analysis effect size (LEfSe)^1^ was used to screen different species between different groups. Linear discriminant analysis value > 2 and *P* < 0.05 were considered statistically significant in LEfSe). Interactions between fecal bacterial species (or between fecal bacterial species and differential expression genes) were assessed by Spearman correlation (R v3.5.1) (Supplementary Figure 1).

### Statistical Analysis

All data were expressed as means ± standard error of the mean. SPSS software (v22.0) and GraphPad Prism software (v8.0) were used for statistical analysis and picture drawing. *P* < 0.05 was considered statistically significant.

### Data Availability

All the sequence data in this study are available in the Sequence Read Archive (SRA) database (SRA accession number of RNA-seq data: SPR305475; SRA accession number of 16S rRNA sequencing data: SPR305592).

## Results

### Phenotypic Characteristics of Mesenchymal Stem Cells

Mesenchymal stem cells were isolated from bone marrow tissues of GFP-transgenic mice and identified by fluorescence microscope and FCM. Spindle-shaped cells were observed, and all the cells expressed green fluorescence visualized by fluorescence microscope (Supplementary Figure 2A); isolated MSCs were confirmed by FCM for positive for CD105, CD73, CD90, CD44, and Sca-1 but negative for CD11b and CD45. Meanwhile, GFP expression was also identified by FCM (Supplementary Figure 2B).

### Mesenchymal Stem Cell Migrated to the Colon and Reduced the Initiation of Colon Tumors Induced by Azoxymethane/Dextran Sulfate Sodium

**FIGURE 1:**
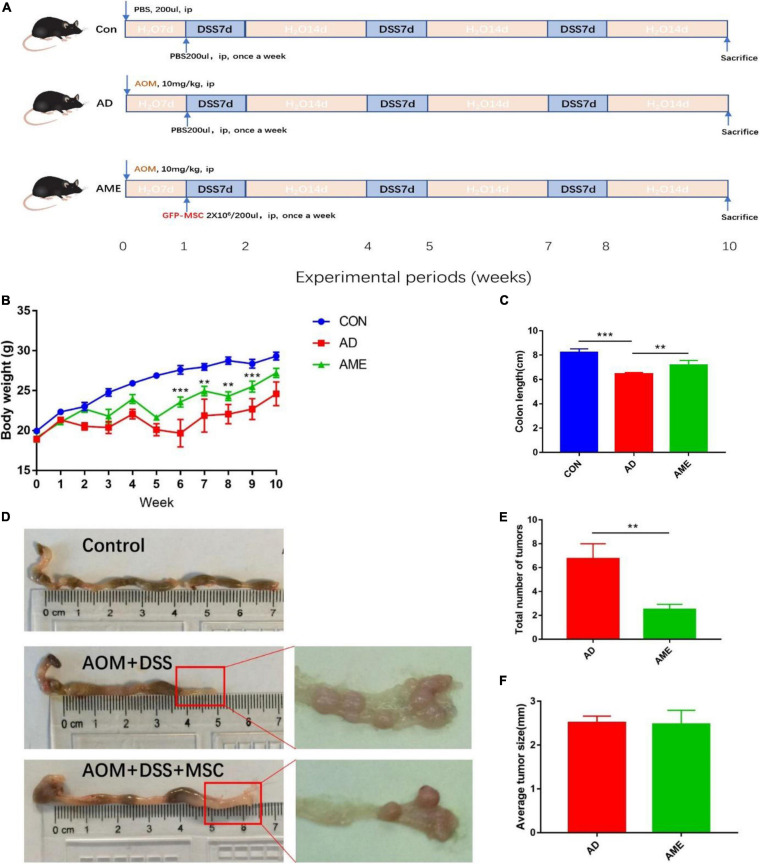
MSCs administration reduced the initiation of colon tumors induced by AOM/DSS in mice. **(A)** Experimental process of AOM/DSS-induced colon tumors and MSC injection procedure. **(B)** Body weight. **(C)** Colon length. **(D)** Representative macroscopic images of colons and tumors. **(E)** Total tumor numbers in each group. **(F)** Average tumor size in each group. Data are expressed as mean ± SEM, **P < 0.01, ***P < 0.001, *n* = 10 mice per group.

To investigate the effect of MSCs on CAC, an AOM/DSS-induced CAC model was administrated with MSCs. The experimental process is shown in Figure 1A. Mice received the administration of AOM and DSS showed significant weight loss and bloody diarrhea. Colon tumors were mainly located from the middle colon to the distal rectum, which is the predominant location of human colorectal cancer. As shown in Figure 1B, the MSC group had less weight loss compared with the AOM/DSS group. In addition, compared with the control group, the AOM/DSS group showed more notable colon shortening. However, MSC significantly prevented colonic shortening induced by AOM/DSS (Figures 1C,D). Furthermore, fewer tumors were found in mice infused with MSCs compared with that in AOM/DSS mice (Figures 1D,E). Interestingly, there was no significant difference in the average tumor size between the two groups (Figure 1F).

**FIGURE 2:**
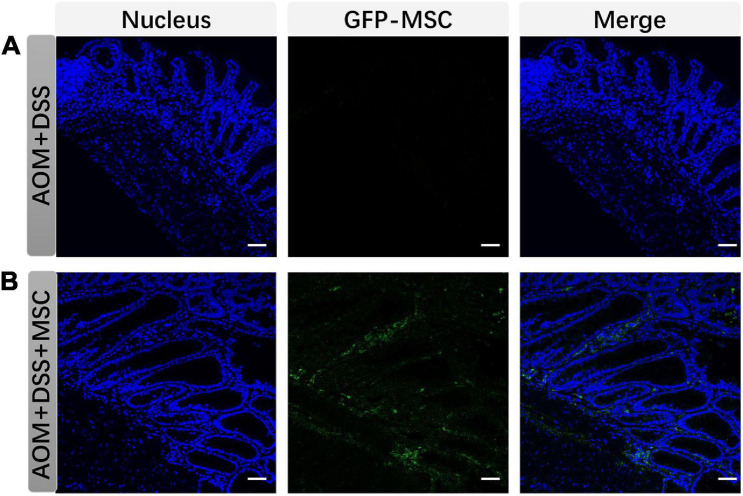
MSCs migrated to colonic tissues. **(A)** and **(B)** show colon tissues stained to explore the migration of MSCs in each group (scare bar, 50 μm).

GFP-MSCs were used to trace its localization in colon tissues. As shown in Figure 2, MSCs were not detected in colonic tissues in AOM/DSS group but can be detected in colonic tissues in the MSC group, which indicated that MSCs migrated to colon tissues when mice had intestinal damage.

**FIGURE 3:**
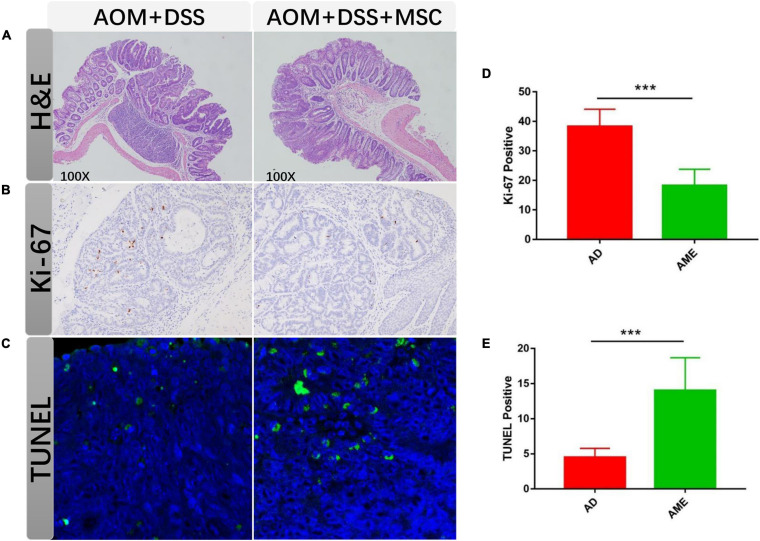
MSC administration regulated tumor cells. **(A)** H&E staining of colon sections in each group. **(B,C)** Ki-67 staining of tumor section in each group. **(D,E)** TUNEL staining of tumor section in each group. Each group randomly selected five tumor tissues and calculated the positive cells. Data are expressed as mean ± SEM, ***P < 0.001.

Colon tissues were stained with H&E, and mucosal damage and colonic inflammation in mice were histologically observed. In the sections of the AOM/DSS group, distorted crypt epithelial, extensive mucosal injury, and massive inflammatory cell infiltration were found. In contrast, the MSC group showed reduced structural disruption and immune cell infiltration compared with the AOM/DSS group (Figure 3A).

Excessive proliferation and apoptosis inhibition have commonly emerged during the development of colon cancer. To detect the effect of MSCs on tumor cells, the proliferation and apoptosis rates in colon tumor tissues were analyzed. Compared with the AOM/DSS group, the positive rate of Ki-67 in the MSC group was significantly augmented (Figures 3B,C). The number of apoptotic cells reduced in the MSC group than that in the AOM/DSS group as assessed by TUNEL. Therefore, the results suggested that MSC infusion promoted apoptosis and inhibited proliferation in intestinal tumor cells (Figures 3D,E).

### Differential Expression of Genes in Intestinal Tumor Tissues After Mesenchymal Stem Cell Injection

**FIGURE 4:**
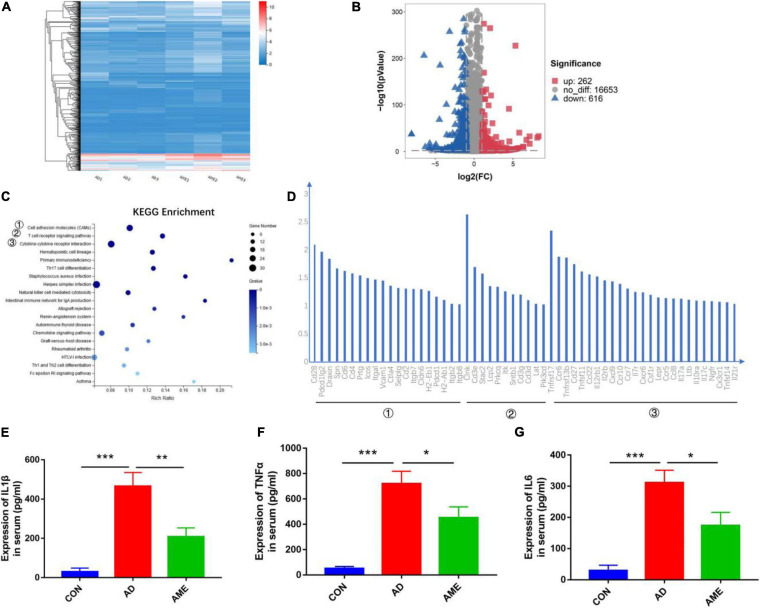
Differential expression of genes in intestinal tumor tissues. **(A)** Hierarchical clustering analysis was performed according to the FPKM of each sample. **(B)** Volcano plot of DEGs in MSC group compared with AOM/DSS group. **(C)** KEGG enrichment analysis was conducted for downregulated genes in MSC group compared with AOM/DSS group. **(D)** Genes in the three top signaling pathways. **(E–G)** Expression of TNF-α, IL-1β, and IL-6 in serum was detected by enzyme-linked immune sorbent assay. *P < 0.05, **P < 0.01, ***P < 0.001. *n* = 3 mice per group.

RNA-seq analysis was performed to acquire the global transcriptomic profiles to compare the differences of tumor transcriptome between the AOM/DSS group and MSC group. The quality of each sample was shown in Additional File 1 and Supplementary Figure 3. The global transcriptomic profiles of FPKM of each sample, fold change, and *Q* value of AD and AME groups were presented in Additional File 2. Hierarchical clustering analysis was performed according to the FPKM of each sample. As shown in Figure 4A, the samples of AOM/DSS mice were clustered into the AD group, whereas the samples of MSCs injection mice were clustered into the AME group. Differential expression gene analysis was performed, and 878 DEGs were obtained in the MSC group compared with that of the AOM/DSS group (262 upregulated genes and 616 downregulated genes) (Figure 4B). Subsequently, we used the KEGG to analyze the biological signaling pathways involved in the 616 downregulated genes. As shown in Figures 4C,D, KEGG enrichment analysis revealed that “cell adhesion molecules,” “T cell receptor signaling pathway,” and “cytokine–cytokine receptor interaction” were the three top signaling pathways involved (Additional File 3). Thus, RNA-seq analysis helps to elucidate that immune responses participate in the mechanism of MSCs to reduce tumor initiation.

Chronic inflammation is the key driver of the CAC; the RNA-seq transcriptome study demonstrated that MSCs reduce tumor initiation through immune responses, so we detected pro-inflammatory cytokines TNF-α, IL-1β, and IL-6 in serum to determine the systematic immune responses. As shown in Figures 4E–G, the expressions of cytokines in mice sera were lower in the MSC group compared with that of the AOM/DSS group.

### Mesenchymal Stem Cells Altered the Composition of Intestinal Flora in Mice

**FIGURE 5:**
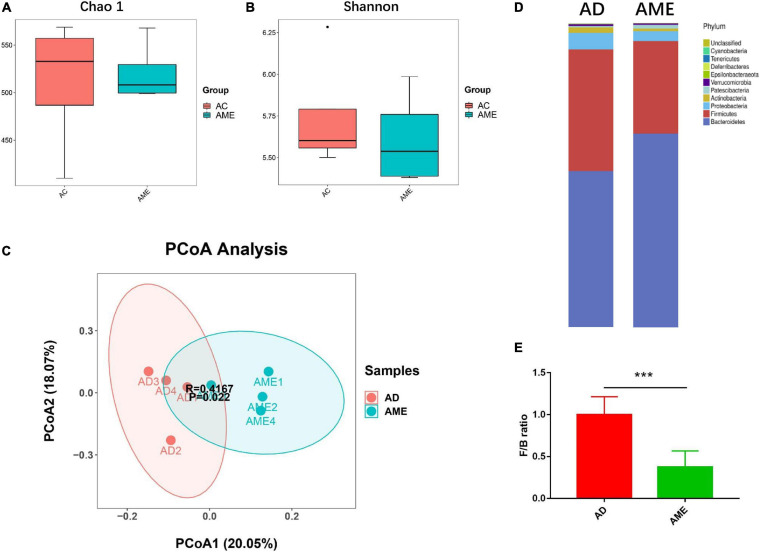
MSCs altered the composition of intestinal flora in mice. **(A,B)** α-Diversity measured by Chao1 and Shannon indexes (*P* > 0.05). **(C)** β-diversity measured by PCoA based on unweighted UniFrac distance and ANOSIM’s analysis. **(D)** Relative bacterial abundance at phylum levels. **(E)** Firmicutes to Bacteroidetes ratio. Data are expressed as mean ± SEM, ***P < 0.001, *n* = 4 mice per group.

It has been reported that MSCs could regulate gut microbiome dysbiosis in mouse colitis model, so we predicted that MSCs could also change the intestinal flora of CAC mice induced by AOM/DSS. Fecal contents of killed mice were collected, and 16S rRNA sequencing was conducted to explore the effect of MSCs on the gut microbiota of CAC mice. α-Diversity of the gut microbiome was not affected by MSCs, which is evaluated by Chao1 and Shannon indexes, respectively (Figures 5A,B). β-Diversity, assessed by PCoA of the unweighted and ANOSIM, showed that the two groups were clearly clustered into two separate groups (Figure 5C). In summary, we found a dissimilarity of gut bacteria between the AOM/DSS and MSC groups.

As shown in Figure 5D, there are 10 major phyla present in the gut microbiota, both in the AOM/DSS and MSC groups, among which the phylum Bacteroidetes was most predominant. We then analyzed the ratio of Firmicutes/Bacteroidetes, which is usually increased in CAC patients. We observed a reduction of Firmicutes/Bacteroidetes ratio in the MSC group compared with the AOM/DSS group (Figure 5E), which means this feature in CRC could be reversed by MSC administration.

**FIGURE 6:**
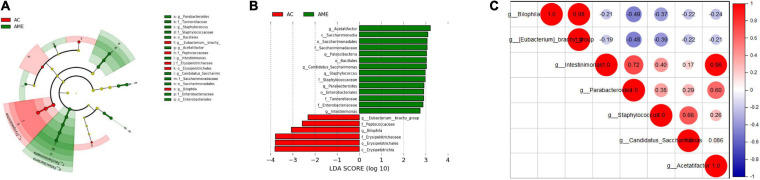
Fecal microbial community altered after MSCs administration. **(A,B)** Significant differences of bacteria between the two groups. **(C)** Correlation between different bacteria at genus level. *n* = 4 mice per group.

Furthermore, LEfSe was performed to elucidate the specific changes in bacterial taxa after MSC injection. At the genus level, a higher abundance of *Staphylococcus*, *Candidatus Saccharimonas*, *Acetatifactor*, *Intestinimonas*, and *Parabacteroides* and a lower abundance of *Bilophila* and *Eubacterium brachy* were detected in the MSC group (Figures 6A,B and Additional File 4). Interactions between these genera were then explored using correlation analysis. There was a significant positive relationship between *Biophilia* and *E. branchy* and between *Acetatifactor* and *Intestinimonas* (Figure 6C).

### Correlations Between the Bacteria and Differential Expression Genes

**FIGURE 7:**
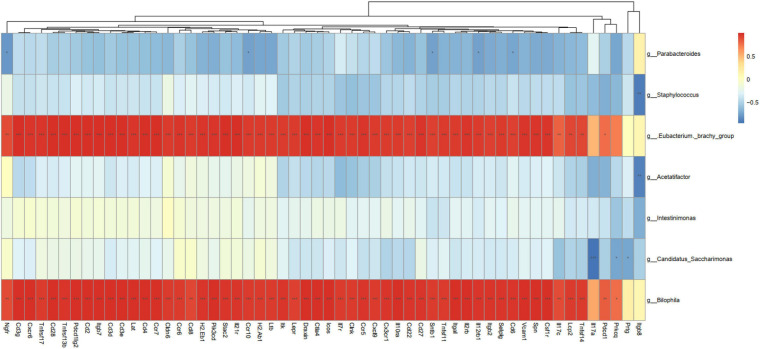
Correlation analysis between differentially expressed genes and bacterial abundance. *P < 0.05, **P < 0.01, ***P < 0.001, *n* = 3 mice per group.

Correlation analysis of the seven bacterial genera and 56 DEGs with three signaling pathway parameters was further conducted to analyze the association between gut microbiota and tumor transcriptome. As shown in Figure 7, *Bilophila* and *E. brachy* had a significant positive correlation with immune parameters, which indicated that these two genera could promote colitis and tumorigenesis through regulate immunity.

## Discussion

Chronic inflammation has been considered as the key driver of CRC (Keum and Giovannucci, 2019), and IBD patients have an increased risk of developing CAC (Chumanevich et al., 2010; Jess et al., 2012). In current years, MSCs have been used in preclinical studies in rodent models and clinical trials in humans to treat IBD and have shown considerable promising results (Liang et al., 2011; Anderson et al., 2013; Park et al., 2015; Lee et al., 2016; Cao et al., 2017). Previous studies also showed that MSCs could migrate to the colon and inhibit CAC. Nasuno et al. (2014) noted that MSCs could reduce the tumor initiation, whereas WNT and TGF-β-Smad signaling pathways were dysregulated in subsequent carcinogenesis, Tang et al. (2015) proved that the differentiation of Treg through Smad2 could be induced by MSCs to suppress the development of CAC, and Chen et al. (2014) determined that MSCs might attenuate the carcinogenesis by reducing pro-inflammatory cytokines release and STAT3 activation. Besides immunological regulation function, MSCs have also been proven to ameliorate gut microbiota dysbiosis (Soontararak et al., 2018), which is considered as another important regulator in the initiation and progression of CRC. Here, our central finding is that MSC administration diminished the initiation of colon tumors in mice through regulating inflammatory status and gut microbiota dysbiosis.

Colitis-associated colon cancer mouse model induced by AOM/DSS mimics the pathological process of human colitis-associated colorectal cancer and has been proven to be valuable in predicting the efficacy of chemical prevention in humans (De Robertis et al., 2011). The significant advantages of the AOM/DSS mouse model are that factors influencing tumor initiation should lead to changes in tumor numbers, whereas factors affecting tumor progression should lead to changes in the average size (Nasuno et al., 2014). Our results found that bone marrow-derived MSC injection significantly reduced the average tumor number per mouse without affecting the average tumor diameter, thus suggesting that MSCs could reduce tumor initiation induced by AOM/DSS rather than tumor progression.

Chronic inflammation functions as the key driver of CAC through inducing gene mutations, promoting proliferation, and reducing apoptosis. In the current experiment, less weight loss, longer colon length, reduced tumor numbers, decreased rate of positive Ki67, and increased rate of apoptotic cells were detected after MSC injection. Furthermore, RNA-seq was performed in colonic tumor tissues, and in the MSC group, immune-associated pathways “cell adhesion molecules,” “T cell receptor signaling pathway,” and “cytokine–cytokine receptor interaction” were significantly reduced. Furthermore, we detected pro-inflammatory cytokines TNF-α, IL-1β, and IL-6 in serum and found that MSCs could reduce the release of pro-inflammatory cytokines in AOM/DSS-induced chronic inflammation. These results together suggest that MSCs can attenuate tumorigenesis by inhibiting chronic inflammation.

A potential secondary mechanism of MSCs involves microbiome alterations. In this study, the administration of MSCs did not change α-diversity but change β-diversity of the gut microbiome, indicating that MSC injection cannot change the abundance but can significantly change diversity. Furthermore, we identified 10 predominant phyla in the gut microbiome: Firmicutes, Bacteroides, Proteobacteria, Actinobacteria, Patescibacteria, Verrucomicrobia, Epsilonbacteraeota, Deferribacteres, Tenericutes, and Cyanobacteria. Among them, Firmicutes and Bacteroides are the two most predominant bacterial phyla. Previous studies suggested that a low Firmicutes/Bacteroides ratio signifies a healthy condition, whereas an increasing ratio of Firmicutes/Bacteroides was observed in CRC patients. Our study demonstrated that MSC treatment led to a significant decrease in the Firmicutes/Bacteroides ratio.

The analysis in genus levels showed an injection of MSCs that increased the abundance of potentially beneficial bacteria and decreased the abundance of potentially harmful bacteria in the gut microbiome of mice. Notably, the abundance of *Parabacteroides*, *Staphylococcus*, *Acetatifactor*, *Intestinimonas*, and *Candidatus Saccharimonas* was increased after MSC administration. *Parabacteroides* in feces was proven to inversely associate with colonic tumor numbers and has anti-inflammatory and anticancer properties (Koh et al., 2018). *Staphylococcus* is a commensal strain and reportedly triggers apoptosis (Zhang et al., 2017) and may protect against neoplasia (Nakatsuji et al., 2018). *Acetatifactor*, which is a butyrate bacterium, has been demonstrated to decrease DSS-induced colitis in mice (Kim et al., 2020) and has been proven to be a benefit in anticancer activities (Xu et al., 2020). *Intestinimonas*, another butyrate bacterium, was shown a lower abundance in patients with CRC (Loke et al., 2018) and mice with IBS (Song et al., 2020). A lower abundance of *Candidatus Saccharimonas* has been noted in hypertriglyceridemia-related acute necrotizing pancreatitis in rats (Huang et al., 2017) and high-fat diet-fed mice (Wang et al., 2020), indicating a potential anti-inflammatory role of *Candidatus Saccharimonas*.

Administration of MSCs decreased the level of *E. branchy* and *Bilophila*. *E. branchy* was first isolated from subgingival samples (Vincent et al., 1984) and was proven to stimulate IL-23-related immune responses (Moutsopoulos et al., 2015), which has been identified as a pivotal role in the pathogenesis of IBD and CAC (Neurath, 2019). *Bilophila*, an opportunistic pathogen, has been proven to increase in rodent models of IBD (Cai et al., 2019) and IBD patients (Yilmaz et al., 2018). Taken together, our results showed MSCs could promote beneficial microbiome alterations to cancel colitis-associated tumorigenesis.

It is well-known that intestinal microbiota plays a crucial part in stimulating local immune responses. The protective effect of MSCs may be attributed to the host transcriptome changes mediated by the altered gut microbiome. Based on the RNA sequencing data and 16S rRNA sequencing analysis, we studied the complex interaction between the host transcriptome profile and fecal microbiota. MSC injection can inhibit inflammation and suppress the immune response, augment the abundance of *Intestinimonas* and *Candidatus Saccharimonas*, and decrease the abundance of *Bilophila* and *E. branchy*. Moreover, we found that there is something significantly positive between *Bilophila* and *E. branchy* with the downregulated differential genes, suggesting that these two genera could promote colitis and tumorigenesis through regulate immunity. In accordance with previous studies, *Bilophila* had inherent pro-inflammatory properties (Devkota et al., 2012; Feng et al., 2017), whereas *E. branchy* was proven to stimulate IL-23-related immune responses (Moutsopoulos et al., 2015); however, the contribution and relative importance of *Bilophila*’s pro-inflammatory properties in CAC condition is unknown. More researches are needed in the future to elucidate the contributions of these gut bacteria in the cancer-preventive role of MSCs.

To sum up, this study suggested that the use of MSCs suppressed inflammation, inhibited tumor cell proliferation, and promoted apoptosis in AOM/DSS mice. Furthermore, MSCs did not change the abundance but changed the diversity and composition of the intestinal microbiome, decreased the Firmicutes/Bacteroides ratio, increased the number of the potential beneficial bacterium, and decreased the opportunistic pathogen. Thus, MSCs may be a promising strategy for colitis-associated colorectal cancer.

## Data Availability Statement

The names of the repository/repositories and accession number(s) can be found below: SRA accession number of RNAseq data: PRJNA700873; SRA accession number of 16S rRNA sequencing data: PRJNA700826.

## Ethics Statement

The animal study was reviewed and approved by the Animal Care and Use Committee of Tongji Medical College of Huazhong University of Science and Technology (Permission Number: 2016-0057).

## Author Contributions

RH: conceptualization, methodology, project administration, and manuscript writing. CH: data curation, investigation, and funding acquisition. YL: software and validation. WQ: resources and visualization. XH: conceptualization, funding acquisition, manuscript review, and editing. All authors contributed to the article and approved the submitted version.

## Conflict of Interest

The authors declare that the research was conducted in the absence of any commercial or financial relationships that could be construed as a potential conflict of interest.

## Footnotes

1 http://huttenhower.sph.harvard.edu/galaxy/

## References

[B1] Anderson P. Souza-Moreira L. Morell M. Caro M. O’Valle F. Gonzalez-Rey E. (2013). Adipose-derived mesenchymal stromal cells induce immunomodulatory macrophages which protect from experimental colitis and sepsis. *Gut* 62 1131–1141. 10.1136/gutjnl-2012-302152 22637701

[B2] Bouffi C. Bony C. Courties G. Jorgensen C. Noël D. (2010). IL-6-dependent PGE2 secretion by mesenchymal stem cells inhibits local inflammation in experimental arthritis. *PLoS One* 5:e14247. 10.1371/journal.pone.0014247 21151872 PMC2998425

[B3] Bray F. Ferlay J. Soerjomataram I. Siegel R. L. Torre L. A. Jemal A. (2018). Global cancer statistics 2018: GLOBOCAN estimates of incidence and mortality worldwide for 36 cancers in 185 countries. *CA Cancer J. Clin.* 68 394–424. 10.3322/caac.21492 30207593

[B4] Cai X. Han Y. Gu M. Song M. Wu X. Li Z. (2019). Dietary cranberry suppressed colonic inflammation and alleviated gut microbiota dysbiosis in dextran sodium sulfate-treated mice. *Food Funct.* 10 6331–6341. 10.1039/c9fo01537j 31524900 PMC6800821

[B5] Cao Y. Ding Z. Han C. Shi H. Cui L. Lin R. (2017). Efficacy of mesenchymal stromal cells for fistula treatment of Crohn’s Disease: a systematic review and meta-analysis. *Dig. Dis. Sci.* 62 851–860. 10.1007/s10620-017-4453-x 28168575

[B6] Chen Z. He X. He X. Chen X. Lin X. Zou Y. (2014). Bone marrow mesenchymal stem cells ameliorate colitis-associated tumorigenesis in mice. *Biochem. Biophys. Res. Commun.* 450 1402–1408. 10.1016/j.bbrc.2014.07.002 25010644

[B7] Chiossone L. Conte R. Spaggiari G. M. Serra M. Romei C. Bellora F. (2016). Mesenchymal stromal cells induce peculiar alternatively activated macrophages capable of dampening both innate and adaptive immune responses. *Stem Cells* 34 1909–1921. 10.1002/stem.2369 27015881

[B8] Chow L. Johnson V. Coy J. Regan D. Dow S. (2017). Mechanisms of immune suppression utilized by canine adipose and bone marrow-derived mesenchymal stem cells. *Stem Cells Dev.* 26 374–389. 10.1089/scd.2016.0207 27881051 PMC5327053

[B9] Chumanevich A. A. Poudyal D. Cui X. Davis T. Wood P. A. Smith C. D. (2010). Suppression of colitis-driven colon cancer in mice by a novel small molecule inhibitor of sphingosine kinase. *Carcinogenesis* 31 1787–1793. 10.1093/carcin/bgq158 20688834 PMC2981458

[B10] De Boeck A. Pauwels P. Hensen K. Rummens J. L. Westbroek W. Hendrix A. (2013). Bone marrow-derived mesenchymal stem cells promote colorectal cancer progression through paracrine neuregulin 1/HER3 signalling. *Gut* 62 550–560. 10.1136/gutjnl-2011-301393 22535374

[B11] De Robertis M. Massi E. Poeta M. L. Carotti S. Morini S. Cecchetelli L. (2011). The AOM/DSS murine model for the study of colon carcinogenesis: from pathways to diagnosis and therapy studies. *J. Carcinog.* 10:9. 10.4103/1477-3163.78279 21483655 PMC3072657

[B12] Devkota S. Wang Y. Musch M. W. Leone V. Fehlner-Peach H. Nadimpalli A. (2012). Dietary-fat-induced taurocholic acid promotes pathobiont expansion and colitis in Il10-/- mice. *Nature* 487 104–108. 10.1038/nature11225 22722865 PMC3393783

[B13] Feng Z. Long W. Hao B. Ding D. Ma X. Zhao L. (2017). A human stool-derived Bilophila wadsworthia strain caused systemic inflammation in specific-pathogen-free mice. *Gut Pathog.* 9:59. 10.1006/anae.2000.0340 PMC5657053 29090023

[B14] Flemer B. Lynch D. B. Brown J. M. Jeffery I. B. Ryan F. J. Claesson M. J. (2018). Tumour-associated and non-tumour-associated microbiota in colorectal cancer. *Gut* 67:395. 10.1136/gutjnl-2015-309595 PMC5529966 26992426

[B15] Gao F. Chiu S. M. Motan D. A. Zhang Z. Chen L. Ji H. L. (2016). Mesenchymal stem cells and immunomodulation: current status and future prospects. *Cell Death Dis.* 7:e2062. 10.1038/cddis.2015.327 26794657 PMC4816164

[B16] Hong Y. S. Ahn Y. T. Park J. C. Lee J. H. Lee H. Huh C. S. (2010). 1H NMR-based metabonomic assessment of probiotic effects in a colitis mouse model. *Arch. Pharm. Res.* 33 1091–1101. 10.1007/s12272-010-0716-1 20661720

[B17] Huang C. Chen J. Wang J. Zhou H. Lu Y. Lou L. (2017). Dysbiosis of intestinal microbiota and decreased antimicrobial peptide level in paneth cells during hypertriglyceridemia-related acute necrotizing pancreatitis in rats. *Front. Microbiol.* 8:776. 10.3389/fmicb.2017.00776 28522995 PMC5415626

[B18] Huang W. H. Chang M. C. Tsai K. S. Hung M. C. Chen H. L. Hung S. C. (2013). Mesenchymal stem cells promote growth and angiogenesis of tumors in mice. *Oncogene* 32 4343–4354. 10.1038/onc.2012.458 23085755

[B19] Jess T. Rungoe C. Peyrin-Biroulet L. (2012). Risk of colorectal cancer in patients with ulcerative colitis: a meta-analysis of population-based cohort studies. *Clin. Gastroenterol. Hepatol.* 10 639–645. 10.1016/j.cgh.2012.01.010 22289873

[B20] Jostins L. Ripke S. Weersma R. K. Duerr R. H. McGovern D. P. Hui K. Y. (2012). Host-microbe interactions have shaped the genetic architecture of inflammatory bowel disease. *Nature* 491 119–124. 23128233 10.1038/nature11582 PMC3491803

[B21] Keum N. Giovannucci E. (2019). Global burden of colorectal cancer: emerging trends, risk factors and prevention strategies. *Nat. Rev. Gastroenterol. Hepatol.* 16 713–732. 10.1038/s41575-019-0189-8 31455888

[B22] Kim J. Choi J. H. Ko G. Jo H. Oh T. Ahn B. (2020). Anti-Inflammatory properties and gut microbiota modulation of *Porphyra tenera* extracts in dextran sodium sulfate-induced colitis in mice. *Antioxidants* 9:988. 10.3390/antiox9100988 PMC7602078 33066339

[B23] Koh G. Y. Kane A. Lee K. Xu Q. Wu X. Roper J. (2018). Parabacteroides distasonis attenuates toll-like receptor 4 signaling and Akt activation and blocks colon tumor formation in high-fat diet-fed azoxymethane-treated mice. *Int. J. Cancer* 143 1797–1805. 10.1002/ijc.31559 29696632

[B24] Lee H. J. Oh S. H. Jang H. W. Kwon J. H. Lee K. J. Kim C. H. (2016). Long-term effects of bone marrow-derived mesenchymalstem cells in dextran sulfate sodium-induced murine chronic colitis. *Gut Liver* 10 412–419. 27114436 10.5009/gnl15229 PMC4849695

[B25] Liang L. Dong C. Chen X. Fang Z. Xu J. Liu M. (2011). Human umbilical cord mesenchymal stem cells ameliorate mice trinitrobenzene sulfonic acid (TNBS)-induced colitis. *Cell Transpl.* 20 1395–1408. 10.3727/096368910x557245 21396175

[B26] Lin R. Han C. Ding Z. Shi H. He R. Liu J. (2020). Knock down of BMSC-derived Wnt3a or its antagonist analogs attenuate colorectal carcinogenesis induced by chronic *Fusobacterium nucleatum* infection. *Cancer Lett.* 495 165–179. 10.1016/j.canlet.2020.08.032 32920199

[B27] Liu Y. Han Z. P. Zhang S. S. Jing Y. Y. Bu X. X. Wang C. Y. (2011). Effects of inflammatory factors on mesenchymal stem cells and their role in the promotion of tumor angiogenesis in colon cancer. *J. Biol. Chem.* 286 25007–25015. 10.1074/jbc.m110.213108 21592963 PMC3137074

[B28] Loke M. F. Chua E. G. Gan H. M. Thulasi K. Wanyiri J. W. Thevambiga I. (2018). Metabolomics and 16S rRNA sequencing of human colorectal cancers and adjacent mucosa. *PLoS One* 13:e0208584. 10.1371/journal.pone.0208584 30576312 PMC6303059

[B29] Mele V. Muraro M. G. Calabrese D. Pfaff D. Amatruda N. Amicarella F. (2014). Mesenchymal stromal cells induce epithelial-to-mesenchymal transition in human colorectal cancer cells through the expression of surface-bound TGF-beta. *Int. J. Cancer* 134 2583–2594. 10.1002/ijc.28598 24214914 PMC4338537

[B30] Moutsopoulos N. M. Chalmers N. I. Barb J. J. Abusleme L. Greenwell-Wild T. Dutzan N. (2015). Subgingival microbial communities in leukocyte adhesion deficiency and their relationship with local immunopathology. *PLoS Pathog.* 11:e1004698. 10.1371/journal.ppat.1004698 25741691 PMC4351202

[B31] Nakatsuji T. Chen T. H. Butcher A. M. Trzoss L. L. Nam S. J. Shirakawa K. T. (2018). A commensal strain of Staphylococcus epidermidis protects against skin neoplasia. *Sci. Adv.* 4:eaao4502. 10.1126/sciadv.aao4502 PMC5834004 29507878

[B32] Nasuno M. Arimura Y. Nagaishi K. Isshiki H. Onodera K. Nakagaki S. (2014). Mesenchymal stem cells cancel azoxymethane-induced tumor initiation. *Stem Cells* 32 913–925. 10.1002/stem.1594 24715689

[B33] Neurath M. F. (2019). IL-23 in inflammatory bowel diseases and colon cancer. *Cytokine Growth Fact. Rev.* 45 1–8. 10.1016/j.cytogfr.2018.12.002 30563755

[B34] Park J. S. Yi T. G. Park J. M. Han Y. M. Kim J. H. Shin D. H. (2015). Therapeutic effects of mouse bone marrow-derived clonal mesenchymal stem cells in a mouse model of inflammatory bowel disease. *J. Clin. Biochem. Nutr.* 57 192–203. 10.3164/jcbn.15-56 26566304 PMC4639590

[B35] Rhyu J. J. Yun J. W. Kwon E. Che J. H. Kang B. C. (2015). Dual effects of human adipose tissuederived mesenchymal stem cells in human lung adenocarcinoma A549 xenografts and colorectal adenocarcinoma HT-29 xenografts in mice. *Oncol. Rep.* 34 1733–1744. 10.3892/or.2015.4185 26252638

[B36] Sekiya I. Larson B. L. Smith J. R. Pochampally R. Cui J. G. Prockop D. J. (2002). Expansion of human adult stem cells from bone marrow stroma: conditions that maximize the yields of early progenitors and evaluate their quality. *Stem Cells* 20 530–541. 10.1634/stemcells.20-6-530 12456961

[B37] Shinagawa K. Kitadai Y. Tanaka M. Sumida T. Kodama M. Higashi Y. (2010). Mesenchymal stem cells enhance growth and metastasis of colon cancer. *Int. J. Cancer* 127 2323–2333. 10.1002/ijc.25440 20473928

[B38] Song Y. F. Pei L. X. Chen L. Geng H. Yuan M. Q. Xu W. L. (2020). Electroacupuncture relieves irritable bowel syndrome by regulating IL-18 and Gut microbial dysbiosis in a Trinitrobenzene Sulfonic acid-induced post-inflammatory animal model. *Am. J. Chin. Med.* 48 77–90. 10.1142/s0192415x20500044 31918565

[B39] Soontararak S. Chow L. Johnson V. Coy J. Wheat W. Regan D. (2018). Mesenchymal Stem Cells (MSC) derived from induced pluripotent stem cells (iPSC) equivalent to adipose-derived MSC in promoting intestinal healing and microbiome normalization in mouse inflammatory bowel disease model. *Stem Cells Transl. Med.* 7 456–467. 10.1002/sctm.17-0305 29635868 PMC5980202

[B40] Tang R. J. Shen S. N. Zhao X. Y. Nie Y. Z. Xu Y. J. Ren J. (2015). Mesenchymal stem cells-regulated Treg cells suppress colitis-associated colorectal cancer. *Stem Cell Res. Ther.* 6:71. 10.1186/s13287-015-0055-8 PMC4414289 25889203

[B41] Tsai K. S. Yang S. H. Lei Y. P. Tsai C. C. Chen H. W. Hsu C. Y. (2011). Mesenchymal stem cells promote formation of colorectal tumors in mice. *Gastroenterology* 141 1046–1056. 10.1053/j.gastro.2011.05.045 21699785

[B42] Van Staa T. P. Card T. Logan R. Leufkens H. (2005). 5-Aminosalicylate use and colorectal cancer risk in inflammatory bowel disease: a large epidemiological study. *Gut* 54 1573–1578. 10.1136/gut.2005.070896 15994215 PMC1774734

[B43] Vendramini-Costa D. B. Carvalho J. E. (2012). Molecular link mechanisms between inflammation and cancer. *Curr. Pharm. Des.* 18 3831–3852. 10.2174/138161212802083707 22632748

[B44] Vincent J. W. Falkler W. A. Jr. Heath J. R. III (1984). Eubacterium brachy. Reactivity in in vitro bone resorptive bioassay. *J. Periodontol.* 55 93–97. 10.1902/jop.1984.55.2.93 6423804

[B45] Wang D. DuBois R. N. (2013). The role of anti-inflammatory drugs in colorectal cancer. *Annu. Rev. Med.* 64 131–144. 23020877 10.1146/annurev-med-112211-154330

[B46] Wang J. Li P. Liu S. Zhang B. Hu Y. Ma H. (2020). Green tea leaf powder prevents dyslipidemia in high-fat diet-fed mice by modulating gut microbiota. *Food Nutr. Res.* 64:3672. 10.29219/fnr.v64.3672 PMC7681786 33281537

[B47] Wang J. Wang Y. Wang S. Cai J. Shi J. Sui X. (2015). Bone marrow-derived mesenchymal stem cell-secreted IL-8 promotes the angiogenesis and growth of colorectal cancer. *Oncotarget* 6 42825–42837. 10.18632/oncotarget.5739 26517517 PMC4767474

[B48] Wheat W. H. Chow L. Kurihara J. N. Regan D. P. Coy J. W. Webb T. L. (2017). Suppression of canine dendritic cell activation/maturation and inflammatory cytokine release by mesenchymal stem cells occurs through multiple distinct biochemical pathways. *Stem Cells Dev.* 26 249–262. 10.1089/scd.2016.0199 27842458 PMC6913781

[B49] Widder M. Lützkendorf J. Caysa H. Unverzagt S. Wickenhauser C. Benndorf R. A. (2016). Multipotent mesenchymal stromal cells promote tumor growth in distinct colorectal cancer cells by a beta1-integrin-dependent mechanism. *Int. J. Cancer* 138 964–975. 10.1002/ijc.29844 26356035

[B50] Xu X. Feng X. He M. Zhang Z. Wang J. Zhu H. (2020). The effect of acupuncture on tumor growth and gut microbiota in mice inoculated with Osteosarcoma cells. *Chin. Med.* 15:33. 10.1186/s13020-020-00315-z PMC7140491 32292489

[B51] Yilmaz B. Spalinger M. R. Biedermann L. Franc Y. Fournier N. Rossel J. B. (2018). The presence of genetic risk variants within PTPN2 and PTPN22 is associated with intestinal microbiota alterations in Swiss IBD cohort patients. *PLoS One* 13:e0199664. 10.1371/journal.pone.0199664 29965986 PMC6028086

[B52] Zhang X. Hu X. Rao X. (2017). Apoptosis induced by *Staphylococcus aureus* toxins. *Microbiol. Res.* 205 19–24. 10.1016/j.micres.2017.08.006 28942840

