# Supplementary material for: Cancer-Preventive Role of Bone Marrow-Derived Mesenchymal Stem Cells on Colitis-Associated Colorectal Cancer: Roles of Gut Microbiota Involved
Source: Front Cell Dev Biol. 2021 Jun 4;9:642948. doi: 10.3389/fcell.2021.642948 (PMC8212064; doi:10.3389/fcell.2021.642948)
Supplement: Supplementary file 2 [file Table_2.DOCX]

Supplementary Material

# Supplementary Figures


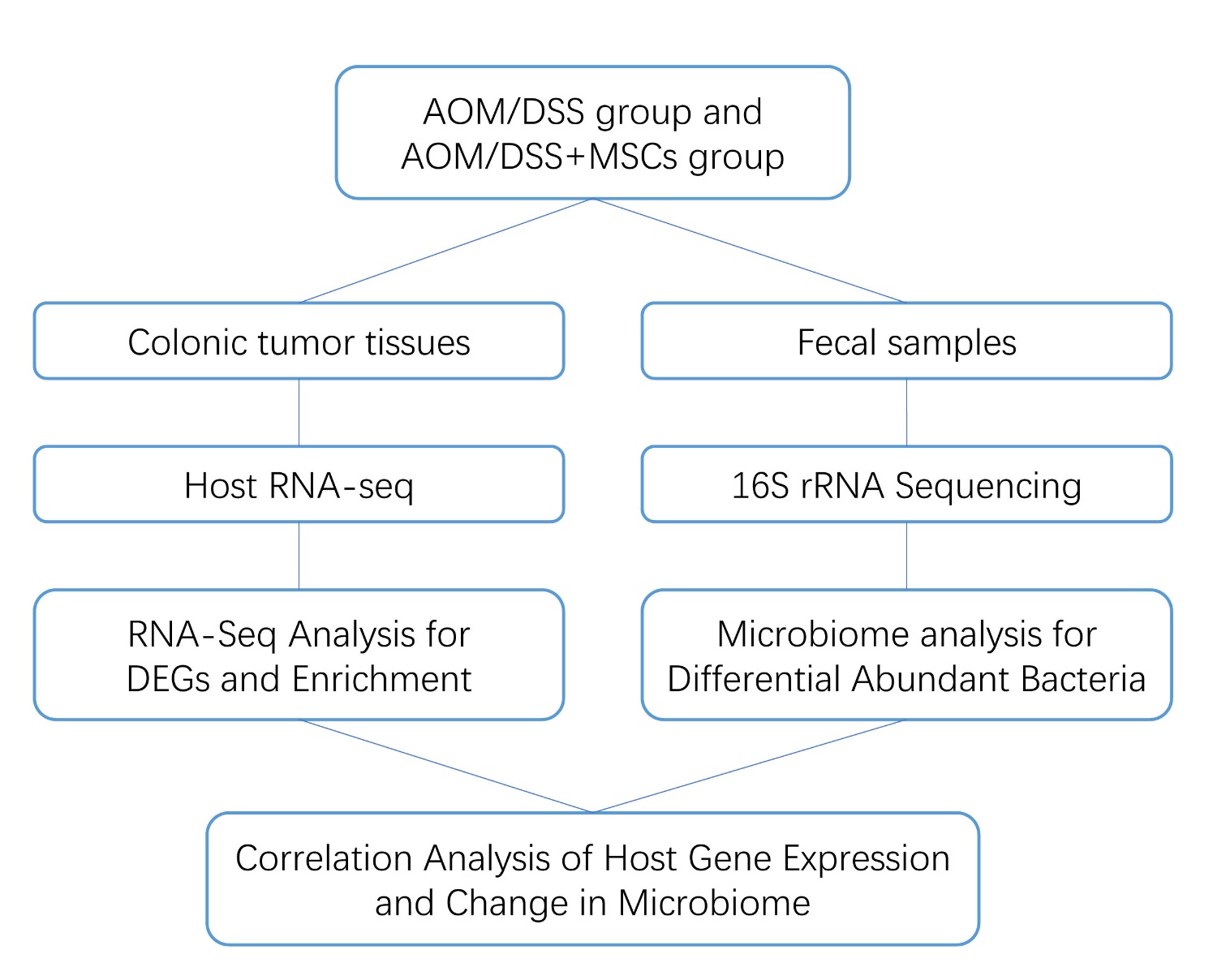


**Supplementary Figure 1.** Experimental pipeline. Flowchart to demonstrate major steps of the study, including sample information, host RNA-Seq analysis, microbiome analysis and integrated analysis.

**
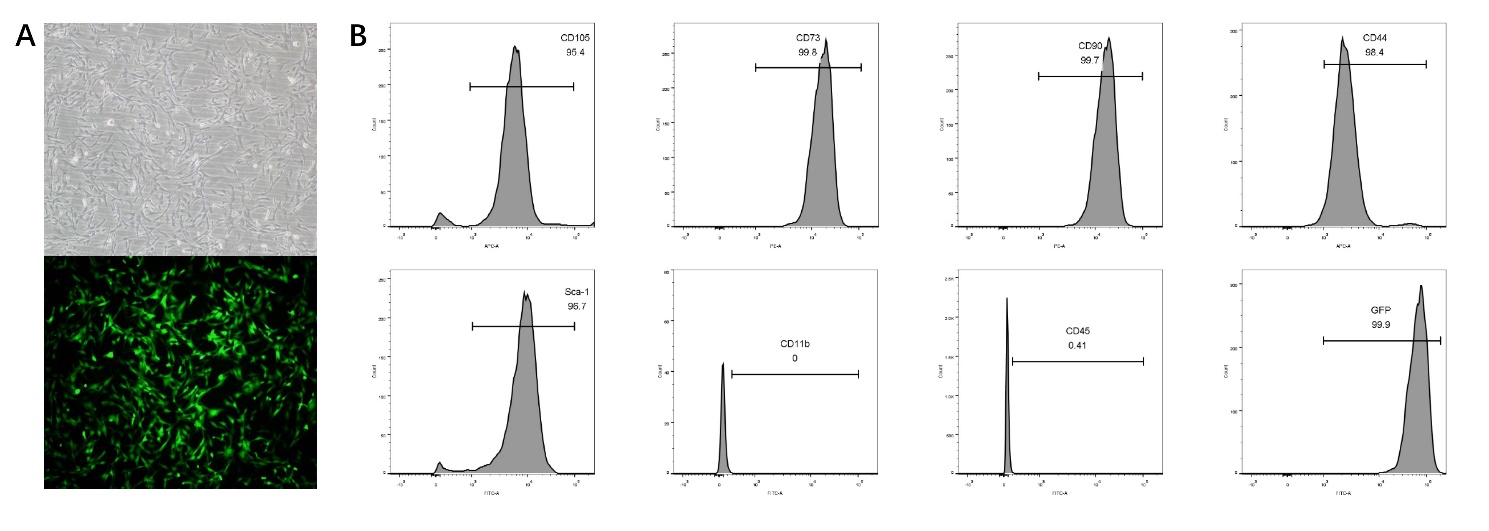
**

**Supplementary Figure 2.** (A) Fluorescence microscope detect the green fluorescent of MSCs. (B) Flow cytometry analysis of surface markers of bone marrow-derived MSCs from C57BL/6 mice, showed that MSCs are positive for CD105, CD73, CD90, CD44, Sca-1, GFP and negative for CD11b, CD45.


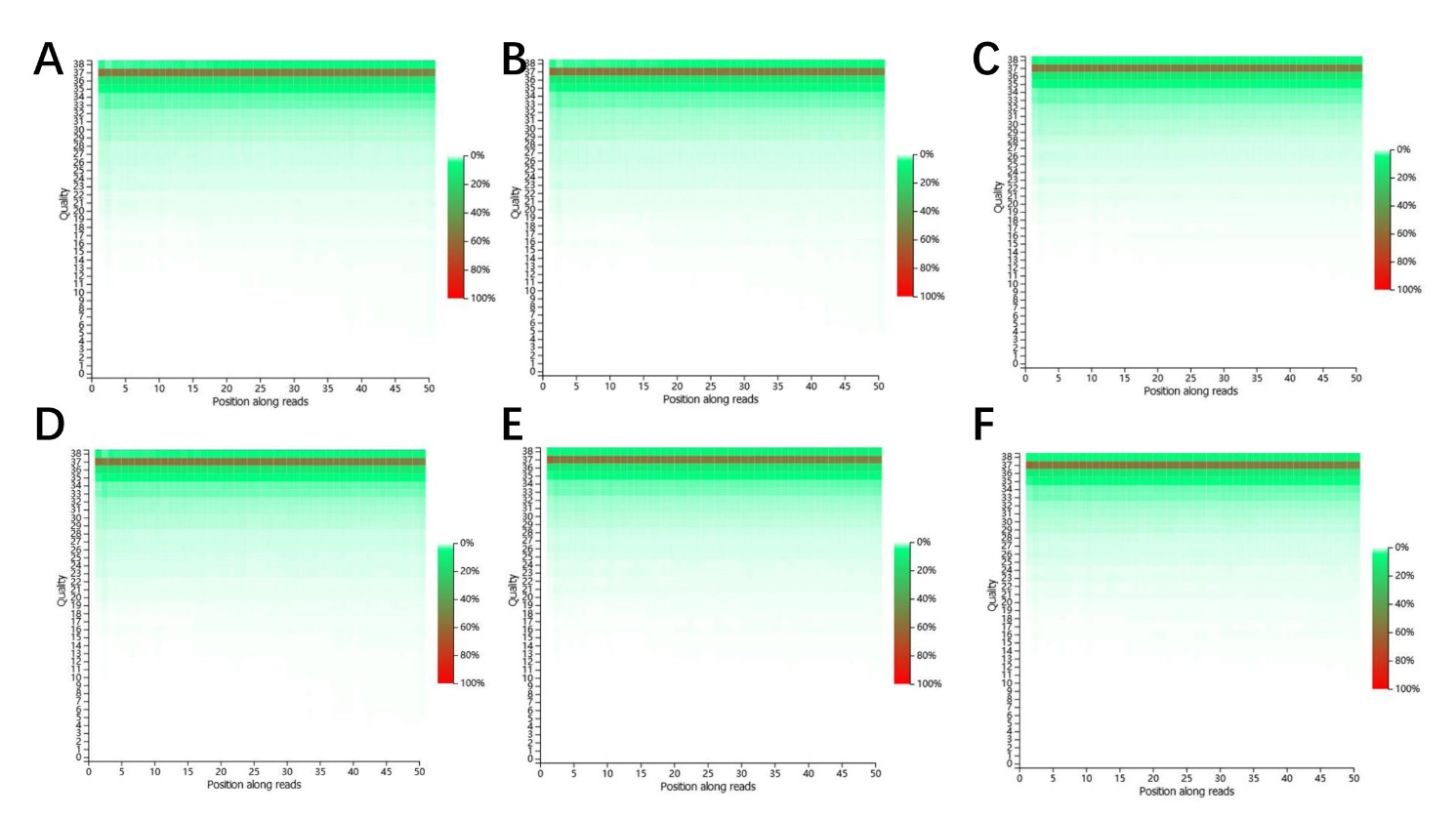


**Supplementary Figure 3.**The distribution map of base quality. X axis represents the location of base in the read, and Y axis represents the quality of base. (A-C) AOM/DSS group, (D-F) AOM/DSS+MSCs group.
